# Effect of SiC Content on the Microstructure and Properties of 6082 Aluminum Matrix Composites

**DOI:** 10.3390/ma19194118

**Published:** 2026-09-26

**Authors:** Wenzhan Huang, Yixuan Chen, Zexin Zhao, Jingkai He

**Affiliations:** School of Materials Science and Engineering, Taiyuan University of Science and Technology, Taiyuan 030024, Chinahejingkai@tyust.edu.cn (J.H.)

**Keywords:** 6082 aluminum alloy, silicon carbide (SiC), stir casting, aluminum matrix composites, mechanical properties

## Abstract

**Highlights:**

• SiC/6082 aluminum matrix composites containing 0–3 wt.% SiC were fabricated by stir casting.
• The mean grain size decreased from 31.279 μm to 17.539 μm as the SiC content increased from 0 to 3 wt.%.
• At 2 wt.% SiC, tensile strength, hardness, and elongation reached their highest measured values of 179.2 MPa, 76.99 HV, and 17.12%, respectively.
• Grain refinement and thermal-mismatch dislocations provide plausible strengthening mechanisms, but the model does not quantify casting-defect losses.
• At 3 wt.% SiC, the mean strength and elongation decreased, while representative SEM images showed pore-like features that may contribute to defect-induced weakening.

**Abstract:**

SiC/6082 aluminum matrix composites containing 0, 0.3, 0.5, 0.7, 1, 2, and 3 wt.% micron-sized SiC were fabricated by stir casting. Optical microscopy and SEM/EDS were used to examine grain structure and local particle-containing regions, while XRD was used for qualitative phase assessment. The recorded mean grain sizes for 0, 0.3, 0.5, 0.7, 1, 2, and 3 wt.% SiC are 31.279, 29.004, 27.919, 23.579, 23.453, 21.797, and 17.539 μm, respectively. Tensile strength and Vickers hardness increased to their highest measured values at 2 wt.% SiC and then decreased at 3 wt.%. Elongation varied non-monotonically but also reached its highest measured value at 2 wt.% SiC. The 2 wt.% composite reached a tensile strength of 179.2 MPa, a hardness of 76.99 HV, and an elongation at break of 17.12%. The comparative XRD patterns are dominated by fcc Al. Open SiC and Si symbols denote reference positions only; weak SiC and Si reflections cannot be reliably distinguished under the present signal-to-noise level and mixed acquisition conditions. The XRD results therefore do not independently confirm a retained SiC phase or a quantitative phase fraction. Grain refinement and thermal-mismatch dislocations provide plausible strengthening mechanisms, whereas the modeled Orowan and load-transfer terms are small at the micron-scale particle size. The lower properties at 3 wt.% coincide with pore-like features in representative SEM fields, but the separate effects of porosity, clustering, and interface integrity remain unquantified.

## 1. Introduction

Aluminum alloys are widely used in aerospace, rail transportation, automotive manufacturing, and defense because of their low density, high specific strength, corrosion resistance, and formability [1,2]. Many structural applications now require lower mass together with reliable performance under demanding service conditions [3,4,5,6]. These requirements can exceed the strength, stiffness, or wear resistance provided by conventional aluminum alloys. 6082 aluminum is a heat-treatable Al-Mg-Si alloy with medium strength, corrosion resistance, and weldability, and it is widely used for structural profiles [7,8,9].

Several approaches have been used to improve 6082 aluminum, friction-stir processing with ceramic or graphite particles, microalloying, and nano-SiC reinforcement [10,11,12,13,14]. Closely related work already includes Al6082/SiC composites containing 0–4 wt.% reinforcement with mechanical and tribological testing [15]. Valappil et al. studied AA6082 reinforced with nano-SiC and Al_2_O_3_ using ultrasonic-assisted and vacuum die stir casting [16], while Kumar et al. examined thermal ageing of bimodal AA6082/TiO_2_/SiC composites produced by stir-squeeze casting [17]. Thus, adding SiC to AA6082 is not itself a new concept. The present study focuses on a single micron-sized reinforcement introduced by conventional stir casting, with closely spaced additions below 1 wt.% and an extension to 3 wt.% to examine the non-monotonic mechanical response. Its contribution is a composition-dependent comparison within this processing route, rather than a claim of superiority over hybrid, nanoscale, or heat-treated composites.

This study examines SiC/6082 aluminum matrix composites containing 0–3 wt.% micron-sized SiC. Phase composition and microstructure are characterized by XRD, optical microscopy, and SEM/EDS; grain size is measured quantitatively; and room-temperature tensile and Vickers hardness tests are used to evaluate mechanical performance. The measured trends are interpreted through grain-refinement, thermal-mismatch, Orowan, and load-transfer models, while also considering the possible weakening effects of particle clustering and pore-like casting defects. The aim is to establish how SiC content affects the balance between strengthening and defect formation in conventionally stir-cast 6082 aluminum composites.

## 2. Experimental Methods

Commercial-grade 6082 aluminum alloy ingots, supplied by Shanxi Huatuo Aluminum Co., Ltd., Yongji, China, were used as the matrix material. The SiC powder was supplied by Weifang Kaihua Silicon Carbide Micropowder Co., Ltd., Weifang, China; the associated thesis material table records a nominal particle size of 7.5 μm and a purity of at least 99.9 wt.%. Table 1 transcribes the chemical composition documented in the associated thesis record; that record does not specify whether the composition originated from a supplier certificate or a separate chemical analysis. The tensile and yield values in Table 1 are the same experimental mean values reported later for the unreinforced control and are not treated as supplier-guaranteed properties.

Samples were labeled 0–6 in order of increasing nominal SiC mass fraction: 0, 0.3, 0.5, 0.7, 1, 2, and 3 wt.%. Metallographic and mechanical test specimens were cut from the same relative position in each ingot. The metallographic specimens (10 mm × 10 mm × 10 mm) were ground and polished, etched in 0.5 vol.% HF for 5–10 s, and examined using an XW-1000 upright optical microscope (China Technology (Tianjin) Instrument Co., Ltd., Tianjin, China). Microstructure and tensile-fracture surfaces were examined using a ZEISS Sigma 300 scanning electron microscope in secondary-electron mode (Carl Zeiss Microscopy GmbH, Jena, Germany), with EDS point and area mapping. Three tensile specimens per composition were prepared by wire electrical-discharge machining to the dimensions shown in Figure 1. Room-temperature tensile tests were performed using a CMT5105 universal testing machine (Sansi Yongheng Technology (Zhejiang) Co., Ltd., Ningbo, China) at a crosshead speed of 0.5 mm/min; the reported tensile values are the means of three parallel tests. Vickers hardness was measured using an HVS-1000 tester (Laizhou Huayin Testing Instrument Co., Ltd., Laizhou, China) at 2.94 N with a 10 s dwell time. Eight positions were tested on each sample; the highest and lowest values were excluded, and the remaining six values were averaged. For grain size analysis, at least five randomly selected optical micrographs per composition were evaluated, with more than 100 grains measured in each image using NanoMeasurer 1.2. These observations are within-specimen measurements and are not independent replicate casting batches. The results are presented descriptively; no statistical significance claim is made because the individual measurements and numerical dispersions are unavailable.

Initial phase characterization used a DX-2700 X-ray diffractometer (Dandong Haoyuan Instrument Co., Ltd., Dandong, China) with Cu Kα radiation, a graphite monochromator, 40 kV tube voltage, 15 mA tube current, a 2θ range of 20–80°, and a scanning rate of 2°/min; phase analysis used MDI Jade 6 software. The comparative patterns in Figure 4 were assembled from the supplied diffraction files and plotted over their common 2θ interval of 20–81°. The Bruker D8 (Bruker AXS GmbH, Karlsruhe, Germany) files record a Cu source, 40 kV, 40 mA, Cu Kα_1_ and Kα_2_ wavelengths of 1.5406 and 1.54439 Å, and an angular increment of approximately 0.020569°. The 0.7 wt.% trace was supplied as a separate 10–90° ASC dataset and was likewise restricted to the common interval. The curves were lightly smoothed, independently normalized, and vertically offset for clarity; their relative peak heights are therefore not used for quantitative phase-fraction comparison. Because acquisition records and detection sensitivity are not identical across all traces, the presence or absence of weak features is not used as proof of a composition-dependent phase appearance, and small peak shifts or line broadening are not interpreted quantitatively.

## 3. Composite Fabrication

Micron-sized SiC particles were used as the reinforcement [18,19]. The particles were oxidized at 1000 °C for 2 h and then held at 300 °C before being added to the melt. This pretreatment was intended to improve wetting by forming a surface SiO_2_ layer that may react locally with liquid aluminum. No untreated-particle control or direct interface characterization was performed; therefore, improved wetting and specific interfacial reaction products are not demonstrated by the present experiments. The 6082 ingots were melted in a graphite crucible in a resistance furnace under flowing argon at 2 L/min. RJ-2 cover flux was applied to the melt surface, and the melt was degassed with dry argon for 10 min before particle addition. The ingots were heated to 780 °C and held for 30 min, followed by a 10 min settling period and slag removal. A stirring head preheated to 200 °C was immersed in the melt and operated at 350 rpm to establish a vortex. The SiC powder was then introduced at an average rate of 4 g/min under continued argon flow. After all particles had been added, stirring was continued at 1000 rpm for 10 min. Surface slag was removed, the melt was cooled to 750 °C, and it was poured into a metal mold preheated to 200 °C and allowed to cool naturally. Composites containing 0.3, 0.5, 0.7, 1, 2, and 3 wt.% SiC were prepared using this procedure. The unreinforced 6082 reference underwent the same melting, argon protection, fluxing, degassing, stirring, cooling, and pouring schedule, except that no SiC powder was introduced. Figure 2 summarizes the experimental workflow.

**Figure 2:**
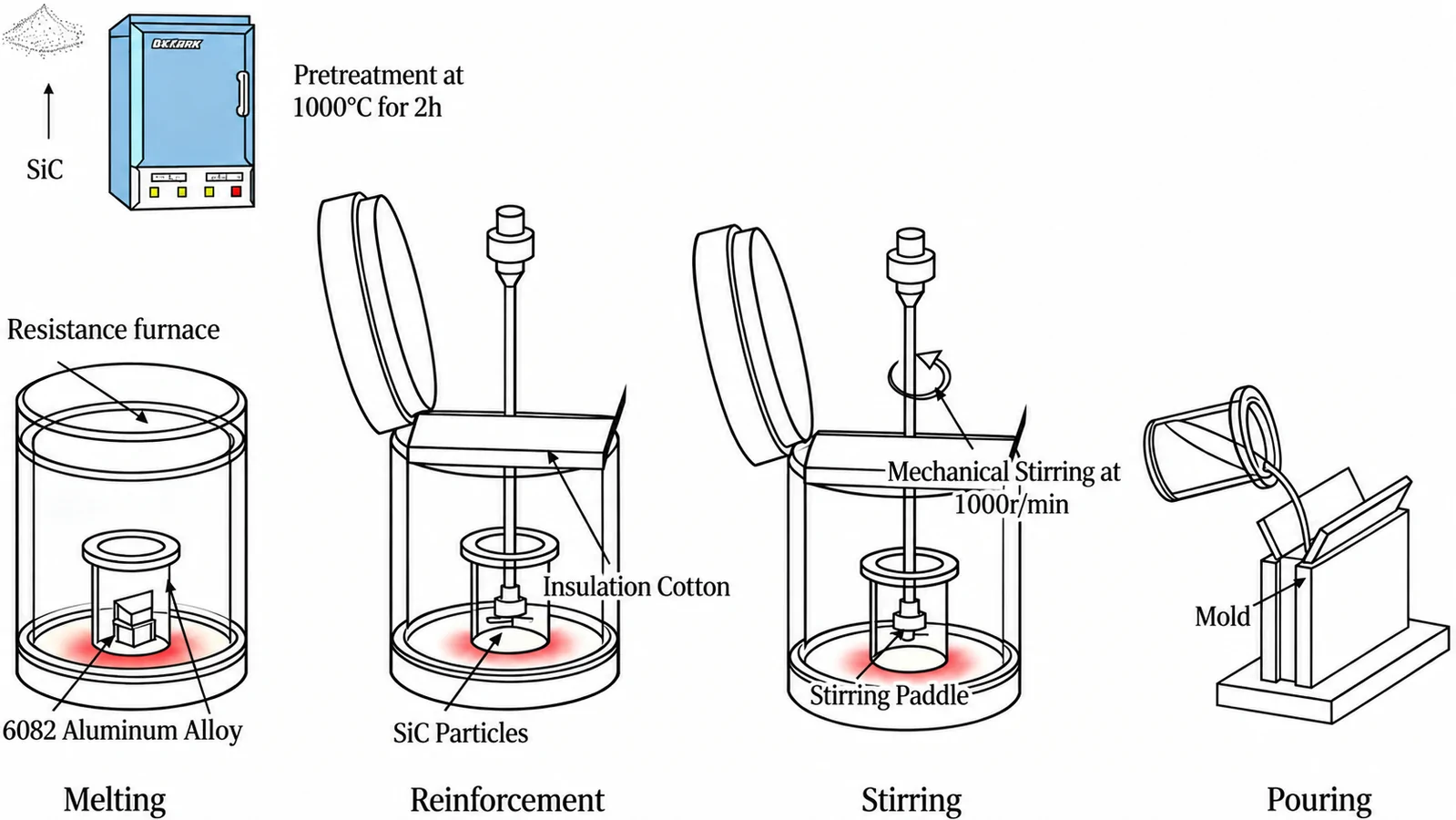
Experimental workflow.

## 4. Results and Discussion

### 4.1. Phase and Microstructural Analysis

Figure 3 shows the XRD pattern of the 0.5 wt.% SiC/6082 aluminum composite. The dominant diffraction peaks are assigned to α-Al, and weaker peaks are labeled as SiC and crystalline Si. The SiC peaks support the incorporation of the reinforcement, while the labeled Si peaks indicate that a separate crystalline Si contribution cannot be excluded. No distinct Al_4_C_3_ diffraction peak was identified within the detection limit of the measurement. This absence does not rule out trace quantities or a thin interfacial reaction layer, so the XRD result is used to identify detectable bulk phases rather than to prove that no interfacial reaction occurred. The mechanical implications of SiC addition are discussed in Section 4.3.

The SiC card numbers cited in Reference [20] are reproduced from a certificate for a separate commercial SiC product and are used only as reference identifiers; they do not establish the polytype or phase purity of the powder used in the present experiments [21].

The raw Al maxima of the 0.5 wt.% trace occur at approximately 38.59°, 44.83°, 65.17°, and 78.29°, within about 0.03–0.12° of the tabulated Cu Kα_1_ positions [22]. The corresponding 0 wt.% trace differs from these positions by no more than approximately 0.04°. These differences are comparable to the angular increment and may also reflect an unresolved Kα doublet, instrumental zero offset, specimen displacement, alloying, and spectral overlap. Without an internal calibration standard and an instrumental broadening standard, the data cannot establish a change in Al lattice parameter, coherent-domain size, or microstrain. No numerical crystallite size or microstrain is inferred.

Figure 4 compares the XRD patterns of unreinforced 6082 and the composites containing 0.3–3 wt.% SiC. The patterns are vertically offset and independently normalized to make weak diffraction features visible; their vertical separation and peak heights should not be compared quantitatively.

**Figure 4:**
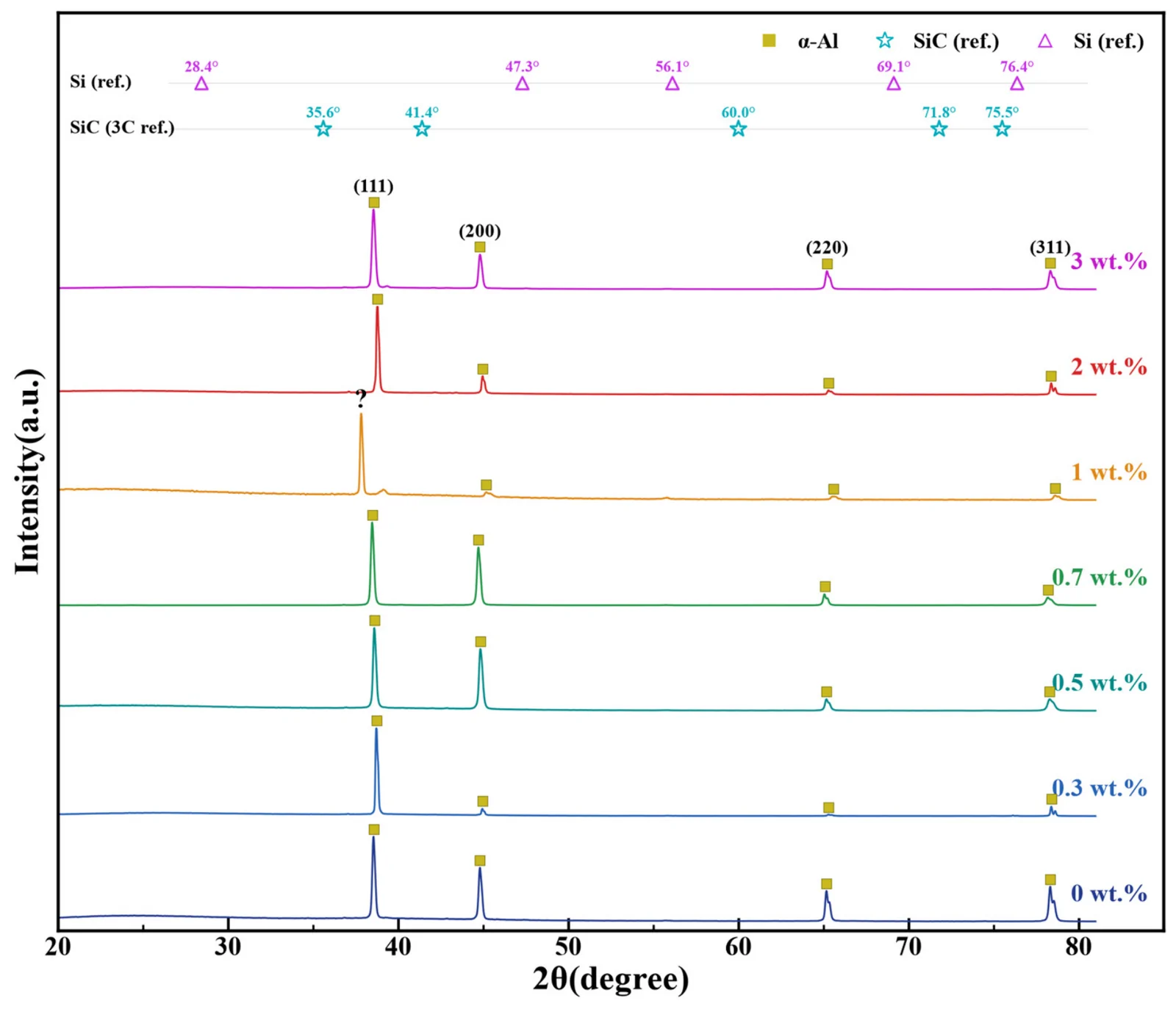
Comparative XRD patterns of unreinforced 6082 and SiC_p_/6082Al composites containing 0, 0.3, 0.5, 0.7, 1, 2, and 3 wt.% nominal SiC.

All seven traces are dominated by the characteristic α-Al reflections. The open SiC and Si symbols in Figure 4 mark reference positions only. Under the present signal-to-noise level, peak overlap, and mixed acquisition records, weak reflections from retained SiC or crystalline Si cannot be reliably distinguished from the background or from neighboring Al features. The approximately 37.8° maximum in the 1 wt.% trace remains unassigned. The comparison therefore does not establish a composition-dependent phase appearance, retained SiC fraction, SiC polytype, or bulk dispersion. No distinct Al_4_C_3_ reflection is resolved, but this observation does not exclude trace quantities or a thin interfacial reaction layer.

Figure 5a–g show optical micrographs of the unreinforced alloy and the composites containing 0.3, 0.5, 0.7, 1, 2, and 3 wt.% SiC, respectively. The 0 wt.% image provides a direct matrix reference for comparing grain morphology and dark features. Optical contrast alone cannot distinguish SiC from intermetallic constituents, pores, or preparation artifacts, so grain size changes are evaluated from the measurements summarized in Figure 6 rather than from contrast alone.

Figure 6 summarizes the grain size measurements from at least five randomly selected optical micrographs per composition, with more than 100 grains evaluated in each image. The mean grain sizes documented in the associated thesis record are 31.279, 29.004, 27.919, 23.579, 23.453, 21.797, and 17.539 μm for 0, 0.3, 0.5, 0.7, 1, 2, and 3 wt.% SiC, respectively. The largest adjacent decrease occurs between 0.5 and 0.7 wt.% (4.340 μm), whereas the difference between 0.7 and 1 wt.% is only 0.126 μm. Heterogeneous nucleation and restriction of grain growth are plausible contributions [23], but the activation of individual nucleation sites was not measured. Because the individual grain measurements and numerical dispersions are unavailable, these exact reported means do not establish that every adjacent composition differs significantly.

Figure 7a shows a bright, irregular region in the 2 wt.% composite. The Si and C signals overlap locally in the corresponding EDS maps, which is consistent with a Si- and C-containing region. Elemental co-location does not determine a Si:C atomic ratio, resolve a thin reaction layer, or distinguish a single particle from a small cluster. The image contrast has not been established as backscattered-electron compositional contrast. Accordingly, the annotation identifies a candidate Si- and C-containing region rather than a definitively identified SiC particle, and this field is not used to prove bulk dispersion uniformity or to exclude Si, carbon-containing phases, or Al_4_C_3_.

Figure 8 presents representative SEM fields for the unreinforced alloy and the composites containing (a) 0, (b) 0.3, (c) 0.5, (d) 0.7, (e) 1, (f) 2, and (g) 3 wt.% SiC. The 0 wt.% image provides a matrix reference for judging particle-containing and pore-like features. The revised annotations identify candidate particle-containing regions rather than confirmed SiC particles; secondary-electron contrast alone does not establish their phase identity. The labels do not determine particle number density, interparticle spacing, or bulk dispersion uniformity. At 3 wt.% SiC, pore-like regions and an irregular particle-rich feature suggest that local heterogeneity may become important. Increased solid loading can hinder melt flow and feeding, while particle addition and stirring may entrain gas or oxide films. However, dark cavities can also include polishing pull-out, and a two-dimensional field cannot establish a bulk pore volume fraction.

### 4.2. Mechanical Properties

Figure 9 shows the mean Vickers hardness obtained from six retained values after testing eight positions and excluding the highest and lowest results for each composition. The recorded means for 0, 0.3, 0.5, 0.7, 1, 2, and 3 wt.% SiC are 60.57, 63.64, 65.37, 66.92, 70.13, 76.99, and 73.82 HV, respectively. Grain refinement and resistance to local plastic flow around hard particles are consistent with the increase [24]. A hardness indentation samples a heterogeneous region of the matrix and reinforcement, so position-to-position scatter is relevant. Because the individual indentation values and numerical standard deviations are unavailable, adjacent means are not interpreted as statistically distinct.

The increase in mean hardness from 0 to 2 wt.% SiC is consistent with the measured grain refinement and with additional barriers to dislocation motion. At 3 wt.% SiC, the lower mean hardness coincides with pore-like regions and a particle-rich feature in the representative SEM image. Pores do not carry indentation load, and local clustering can produce spatially variable constraints; both effects could offset part of the particle-related strengthening. Because the porosity fraction and spatial hardness variation were not quantified, this explanation remains a structure-property interpretation rather than a direct measurement of the defect contribution.

Figure 10 shows one representative engineering stress–strain curve for each SiC_p_/6082Al composition. The unreinforced alloy has a measured mean ultimate tensile strength of 128.33 MPa and a measured mean elongation at break of 7.91%. The mean ultimate tensile strength increases to 179.2 MPa at 2 wt.% SiC, while elongation varies non-monotonically and reaches its highest mean value of 17.12% at 2 wt.% SiC. The recorded mean elongations for 0, 0.3, 0.5, 0.7, 1, 2, and 3 wt.% SiC are 7.91%, 9.42%, 12.17%, 12.73%, 10.24%, 17.12%, and 10.58%, respectively. At 3 wt.% SiC, the mean ultimate tensile strength decreases to 167.41 MPa. The decrease at the highest content is consistent with the pore-like and particle-rich regions observed in representative SEM fields, which may promote stress concentration and earlier crack initiation. Because defect fractions were not quantified, the tensile trend cannot be attributed to porosity alone.

**Figure 10:**
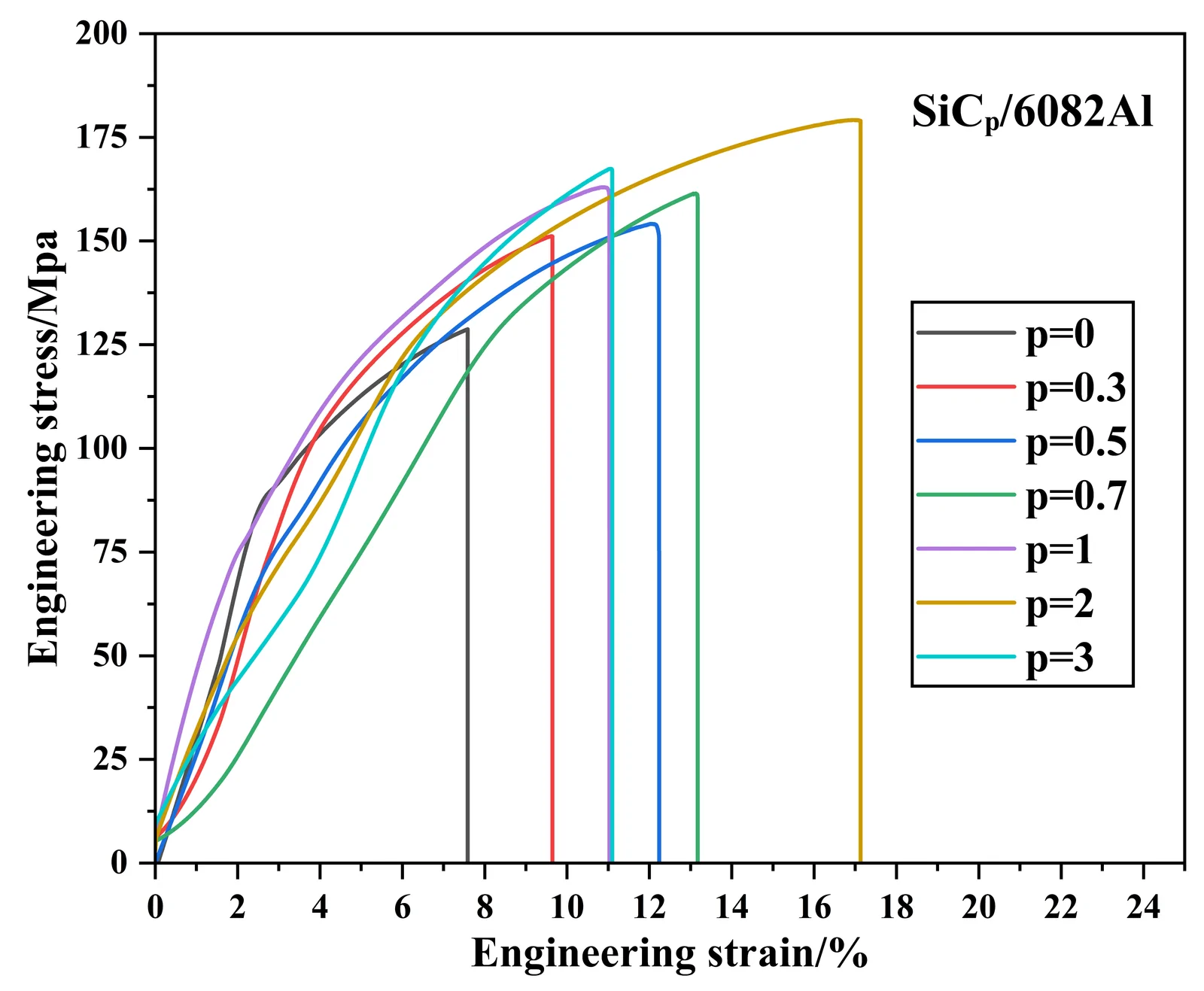
Representative engineering stress–strain curves of SiC_p_/6082Al composites containing 0, 0.3, 0.5, 0.7, 1, 2, and 3 wt.% SiC.

Figure 11 summarizes the tensile strength, yield strength, and elongation at break. The tensile and yield strengths increase from 128.33 and 70.58 MPa, respectively, for the unreinforced alloy to 179.2 and 98.56 MPa at 2 wt.% SiC. Both changes correspond to increases of approximately 39.6%. At 3 wt.% SiC, the tensile and yield strengths decrease to 167.41 and 92.07 MPa.

**Figure 11:**
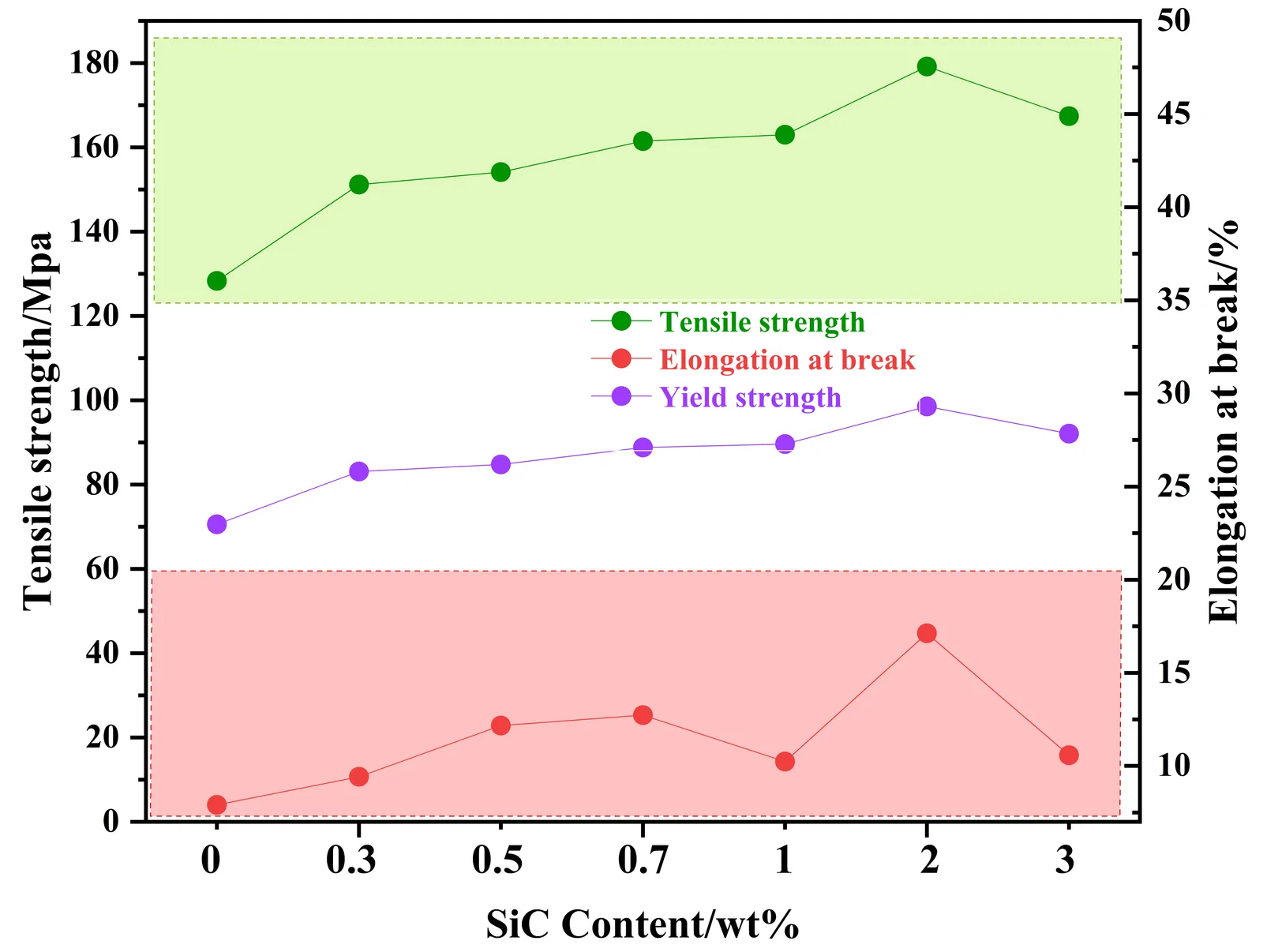
Tensile strength and yield strength (left axis, MPa) and elongation at break (right axis, %) of SiC_p_/6082Al composites as a function of SiC content.

The mechanical property trend reflects a balance between particle-related strengthening and defect-related weakening [25,26]. Up to 2 wt.% SiC, the strength increase is consistent with grain refinement, thermal-expansion mismatch, Orowan resistance, and load transfer. The elongation also reaches 17.12% at 2 wt.% SiC, which is 116.4% above the unreinforced value. This result indicates that the strengthening contribution did not eliminate macroscopic plastic deformation at this composition. At 3 wt.% SiC, all three tensile metrics decrease while pore-like regions and a particle-rich feature appear in a representative SEM field. Such defects can interrupt load transfer and promote local stress concentration. The observed association supports a competition between strengthening and casting defects, but the relative contributions of porosity, clustering, and interfacial debonding cannot be separated without quantitative defect and interface measurements.

The true stress–strain and work-hardening-rate curves in Figure 12 are derived from the representative engineering curves and provide a qualitative comparison of tensile response. Engineering quantities were converted using the conventional uniform-deformation relations before necking, and the work-hardening rate was obtained by numerical differentiation. Conversion to true quantities is applicable only before the onset of necking unless local cross-sectional measurements are available. Because the original processing script and smoothing settings are unavailable, Figure 12 is not used to extract additional fitted parameters or to infer post-necking constitutive behavior.

The plotted work-hardening rate decreases rapidly with strain for the lower SiC additions, while the 2 wt.% curve remains comparatively sustained over a broader interval. Dislocation storage around particles and grain boundaries may contribute to this behavior. The 3 wt.% curve fluctuates more strongly; numerical differentiation can amplify experimental noise, and localized deformation around defects is another possible contribution. Without the raw curves and a documented differentiation procedure, these fluctuations are not taken as direct evidence of a particular defect or dislocation mechanism.

Figure 13 shows tensile-fracture surfaces for the composites containing 0.3, 0.5, 0.7, 1, 2, and 3 wt.% SiC. The 2 wt.% fracture surface contains numerous fine dimples and tear ridges, consistent with ductile fracture and with the maximum measured elongation. At 3 wt.% SiC, the fracture morphology is less uniformly dimpled and is consistent with the lower elongation. The images support a change in fracture morphology with SiC content, but they do not by themselves prove uniform particle dispersion or quantify particle-matrix debonding. The tensile response is therefore interpreted as the combined result of particle-related strengthening, matrix plasticity, and defects that can nucleate cracks.

### 4.3. Strengthening Mechanisms

The yield strength depends on grain size, dislocation density, particle fraction and size, and interface integrity [18,19,27,28,29,30]. Equation (1) combines the load-transfer and Hall–Petch increments additively with a root-sum-square combination of the thermal-mismatch and Orowan terms. It is an idealized strengthening estimate rather than a fitted prediction of the measured yield strengths. The following calculations use the nominal additions and assume complete particle retention, separate particles, and effective interfacial load transfer. These assumptions do not include porosity, clustering, recovery, or debonding losses. For conversion from mass fraction to particle volume fraction, density values of 2.70 g/cm^3^ for the matrix and 3.21 g/cm^3^ for SiC are adopted as model assumptions, not as measured composite densities. The nominal 0.3–3 wt.% additions then correspond to approximately 0.253–2.535 vol.%.

(1) $$∆\sigma ={∆\sigma }_{Load}+{∆\sigma }_{HP}+(({{∆\sigma }_{CTE})}^{2}+{\left({{∆\sigma }_{Orowan})}^{2}\right)}^{\frac{1}{2}}$$

(1) Grain Refinement Strengthening (Hall–Petch)

SiC addition is accompanied by a decrease in the measured mean α-Al grain size. The larger grain-boundary area increases resistance to dislocation motion during tensile deformation [31]. The Hall–Petch contribution to the yield-strength increment is estimated from Equation (2):

(2) $${∆\sigma }_{HP}=K_{y}(d^{-\frac{1}{2}}-d_{0}^{-\frac{1}{2}})$$

Here, Ky is the Hall–Petch coefficient, and d0 and d are the mean grain sizes of the unreinforced matrix and the composite, respectively [32]. For a fixed positive K_y_, the Hall–Petch increment (${∆\sigma }_{HP}$) increases as d decreases. Thus, the continuing decrease in mean grain size at 3 wt.% does not, by itself, explain the lower measured yield strength at that composition. A numerical Hall–Petch series is not reported because a condition-specific Ky and the underlying grain size statistics are needed to establish its magnitude and uncertainty.

(2) Thermal Mismatch Strengthening (CTE)

The coefficients of thermal expansion of SiC and the 6082Al matrix differ substantially. Cooling from the casting temperature can therefore generate thermal strain around the particles and increase the density of geometrically necessary dislocations [33]. The corresponding contribution to the yield-strength increment is estimated from Equations (3), (3a) and (3b):

(3) $${∆\sigma }_{CTE}=\beta G_{m}b\sqrt{\rho^{CTE}}$$

(3a) $$G_{m}=\frac{E_{m}}{2(1+v)}$$

(3b) $$\rho^{CTE}=A\left(\alpha_{m}-\alpha_{p}\right)∆TV_{p}/(bd_{p}(1-V_{p}))$$

The parameters used for this estimate are β = 1.25, A = 12 [34], Poisson’s ratio 0.33, Young’s modulus 70 GPa, and a Burgers vector of 0.286 nm. Equation (3a) gives a matrix shear modulus of approximately 26.3 GPa. The particle diameter of 8.67 μm is the average value adopted in the associated thesis calculation; it differs from the supplier’s nominal size of 7.5 μm and is treated here as a model input because the underlying particle-size distribution is unavailable. The matrix and particle thermal-expansion coefficients are 23.6 × 10^−6^ and 7.8 × 10^−6^ K^−1^, respectively. A temperature interval of 550 K, calculated from the stated 750 °C melt and 200 °C mold temperatures, is retained solely as the idealized input used in the associated model. It is not a measured stress-free cooling interval and does not include cooling to room temperature; therefore, the resulting absolute values should be regarded as illustrative estimates. With the converted volume fractions, Equations (3) and (3b) give thermal-mismatch increments of 3.07, 3.97, 4.70, 5.62, 7.99, and 9.84 MPa for 0.3, 0.5, 0.7, 1, 2, and 3 wt.% SiC. These values neglect recovery and local relaxation and are not measurements of dislocation density or its strengthening contribution.

(3) Orowan Strengthening Mechanism (Orowan)

During tensile deformation, non-shearable SiC particles can impede dislocation motion. When the applied stress reaches the Orowan bypass stress ($\tau_{Orowan}$), dislocations bow around the particles and leave dislocation loops, increasing the resistance to further motion. The estimated Orowan contribution is calculated from Equations (4) and (4a):

(4) $${∆\sigma }_{Orowan}=\frac{0.13G_{m}b}{\lambda }ln\frac{d_{p}}{2b}$$

(4a) $$\lambda =d_{p}[({2V_{p})}^{-\frac{1}{3}}]$$

Using the printed spacing approximation in Equation (4a), the stated particle diameter of 8.67 μm, and the converted volume fractions, the recalculated Orowan increments (∆σOrowan) are 0.22, 0.28, 0.32, 0.37, 0.52, and 0.64 MPa for 0.3, 0.5, 0.7, 1, 2, and 3 wt.% SiC, respectively. The small values result from the micron-scale particle diameter and low volume fractions. Clustering produces a distribution of effective spacings, which this single-spacing estimate does not represent.

(4) Load-Bearing Strengthening (Load)

Load-bearing strengthening represents the direct transfer of part of the applied load from the aluminum matrix to the stiffer SiC particles [35]. Its estimated contribution to the yield-strength increment is calculated using Equation (5):

(5) $${∆\sigma }_{Load}=\frac{1}{2}V_{P}\sigma_{m}$$

Here, $\sigma_{m}$ is the unreinforced matrix yield strength: 70.58 MPa. The calculated ${∆\sigma }_{Load}$ values are 0.09, 0.15, 0.21, 0.30, 0.60, and 0.89 MPa for 0.3, 0.5, 0.7, 1, 2, and 3 wt.% SiC, respectively. This expression assumes an effective interface; incomplete wetting, pores adjacent to particles, or debonding can reduce load transfer. SEM images alone do not measure interfacial shear strength.

The total model increment (∆σ) is not equated to the measured yield-strength difference. Relative to the unreinforced value of 70.58 MPa, the experimental differences at 2 and 3 wt.% are 27.98 and 21.49 MPa, respectively; these are arithmetic differences between reported measurements, not independently calculated sums of strengthening terms. The thermal-mismatch term exceeds the Orowan and load-transfer estimates for the adopted inputs, but its magnitude relative to Hall–Petch strengthening remains unresolved. All three calculated particle-related terms increase with nominal addition, whereas the measured yield strength decreases from 2 to 3 wt.%. This divergence is consistent with additional weakening or relaxation processes omitted by the model. Porosity, clustering, particle pull-out, interface damage, and changes in matrix deformation can contribute, but their separate magnitudes cannot be inferred from the present data.

The principal limitations are the absence of reported uncertainty bars, quantitative pore and cluster distributions, quantitative XRD phase fractions, and direct interface or dislocation characterization. Figure 5 and Figure 8 now include 0 wt.% reference images, providing qualitative matrix controls for the optical and SEM comparisons; however, these images do not by themselves yield porosity fractions or bulk particle-distribution statistics. The observations establish composition-dependent mean trends, but they do not validate a unique strengthening mechanism or demonstrate that every additional SiC increment produces a statistically significant change.

## 5. Conclusions

(1) SiC/6082 aluminum matrix composites containing 0–3 wt.% nominal SiC were fabricated by stir casting. The recorded mean grain size decreases from 31.279 μm for the unreinforced alloy to 17.539 μm at 3 wt.% SiC, with intermediate means of 29.004, 27.919, 23.579, 23.453, and 21.797 μm at 0.3, 0.5, 0.7, 1, and 2 wt.% SiC, respectively. Comparative XRD patterns are dominated by α-Al. The open SiC and Si markers denote reference positions only, and the present scans do not reliably confirm retained SiC, crystalline Si, a SiC polytype, quantitative phase fraction, or dispersion uniformity. SEM/EDS identifies local Si- and C-containing regions that are consistent with, but do not uniquely prove, SiC-containing regions.
(2) The tensile strength, yield strength, and hardness generally increased and reached their highest measured mean values at 2 wt.% SiC, then decreased at 3 wt.%. Elongation varied non-monotonically but also reached its highest measured mean value at 2 wt.% SiC. At 2 wt.% SiC, the tensile strength reached 179.2 MPa, the yield strength reached 98.56 MPa, the hardness reached 76.99 HV, and the elongation at break reached 17.12%.
(3) The fracture-surface observations are consistent with the tensile results. The 2 wt.% composite shows predominantly dimpled ductile-fracture features, whereas the 3 wt.% composite shows a less uniform fracture morphology consistent with reduced ductility. Fracture toughness was not measured, so the results are limited to fracture morphology and tensile elongation.
(4) Grain refinement and thermal-mismatch dislocations are plausible contributors to strengthening. Under the adopted idealized parameters, the thermal-mismatch increment is larger than the Orowan and load-transfer terms, but the relative contribution of Hall–Petch strengthening is not quantitatively established. The lower measured properties at 3 wt.% despite continued grain refinement are consistent with a competition between strengthening and defect-related weakening. Porosity, clustering, and interface damage remain qualitative explanations rather than separately measured strength losses.

## Figures and Tables

**Figure 1:**
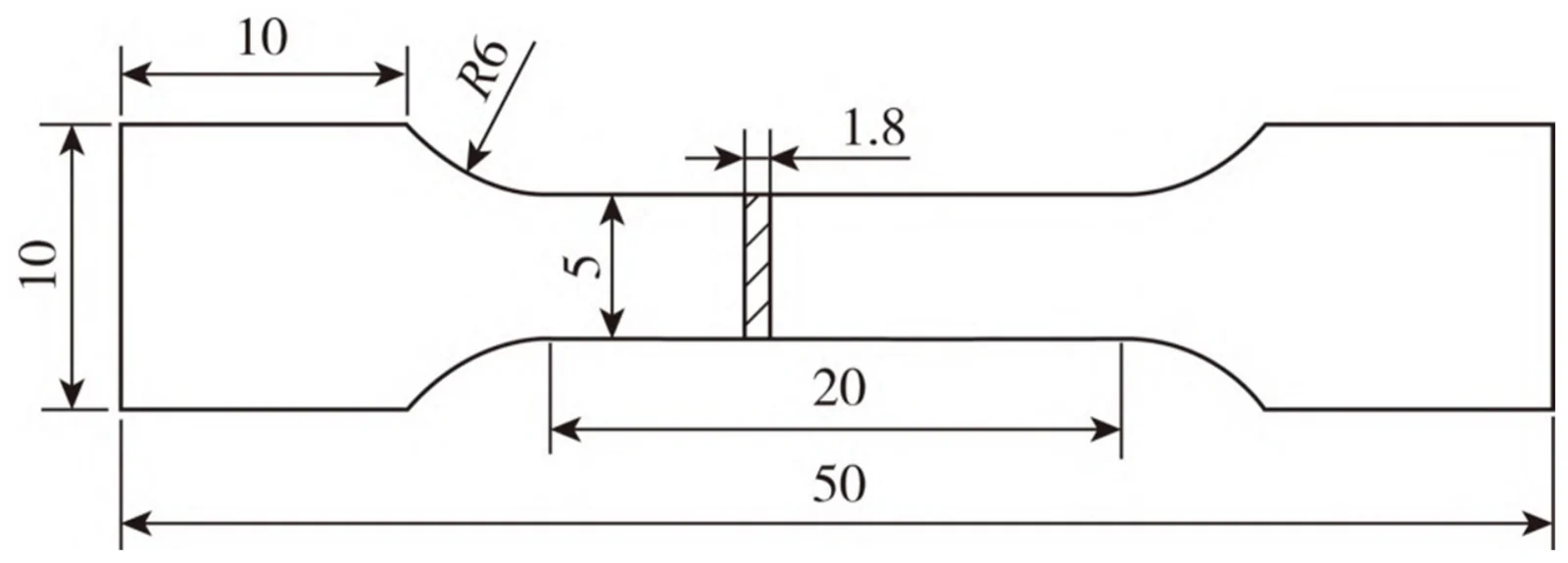
Tensile-specimen geometry (Unit: cm).

**Figure 3:**
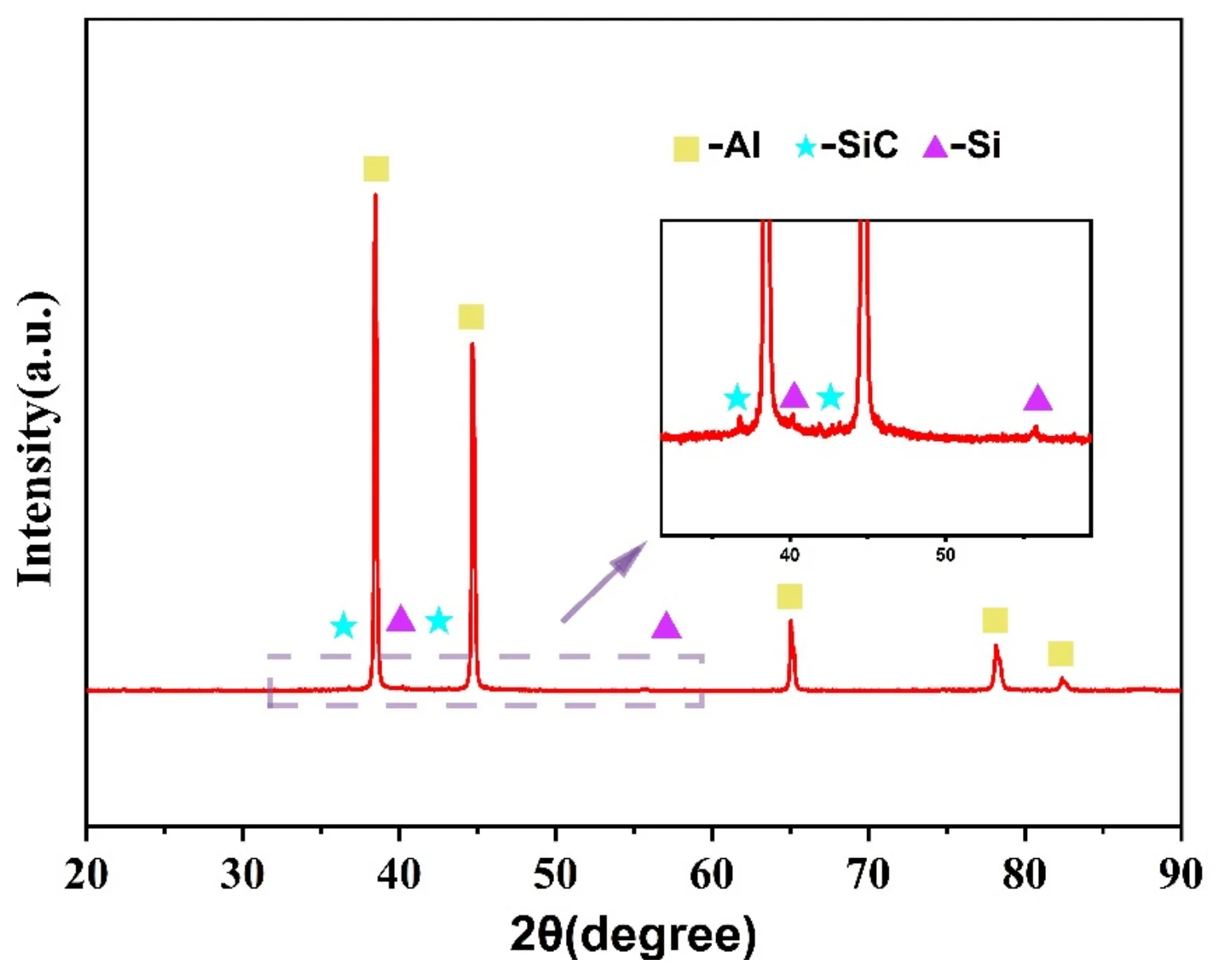
XRD pattern of the 0.5 wt.% SiC_p_/6082Al composite.

**Figure 5:**
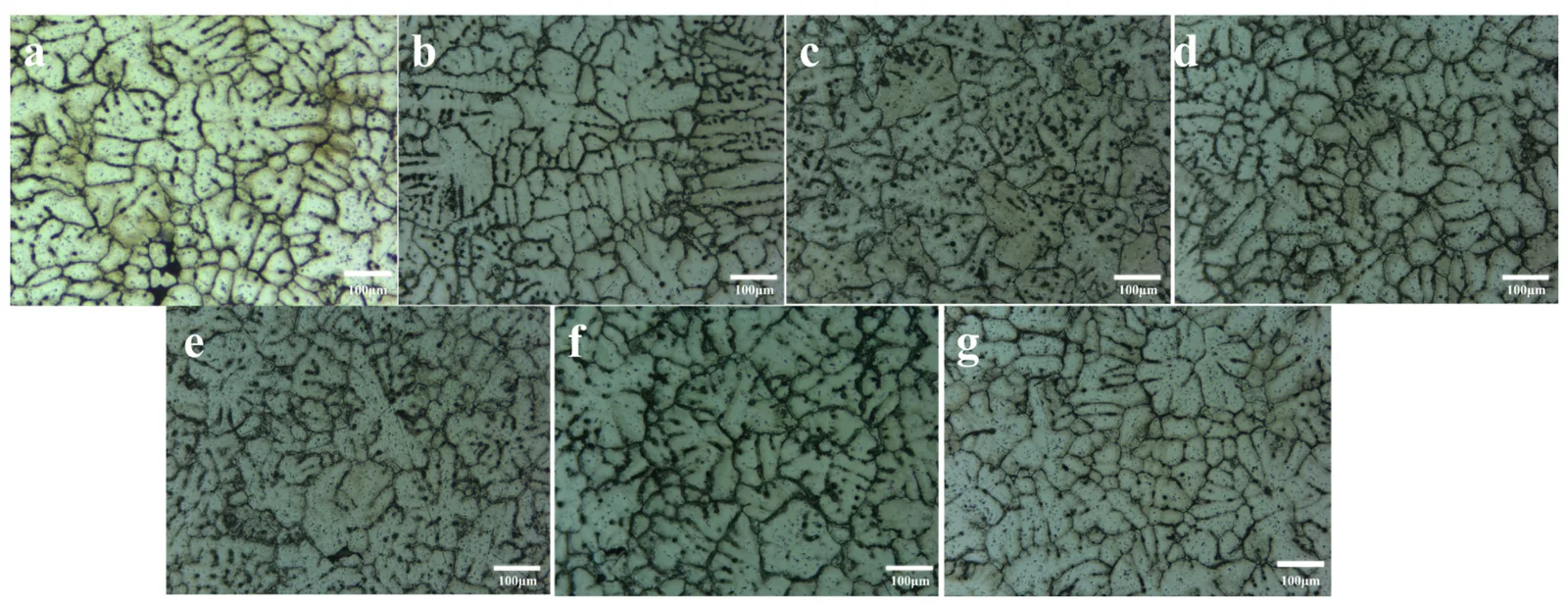
Optical micrographs of 6082 aluminum alloy and SiC_p_/6082Al composites containing (**a**) 0, (**b**) 0.3, (**c**) 0.5, (**d**) 0.7, (**e**) 1, (**f**) 2, and (**g**) 3 wt.% SiC.

**Figure 6:**
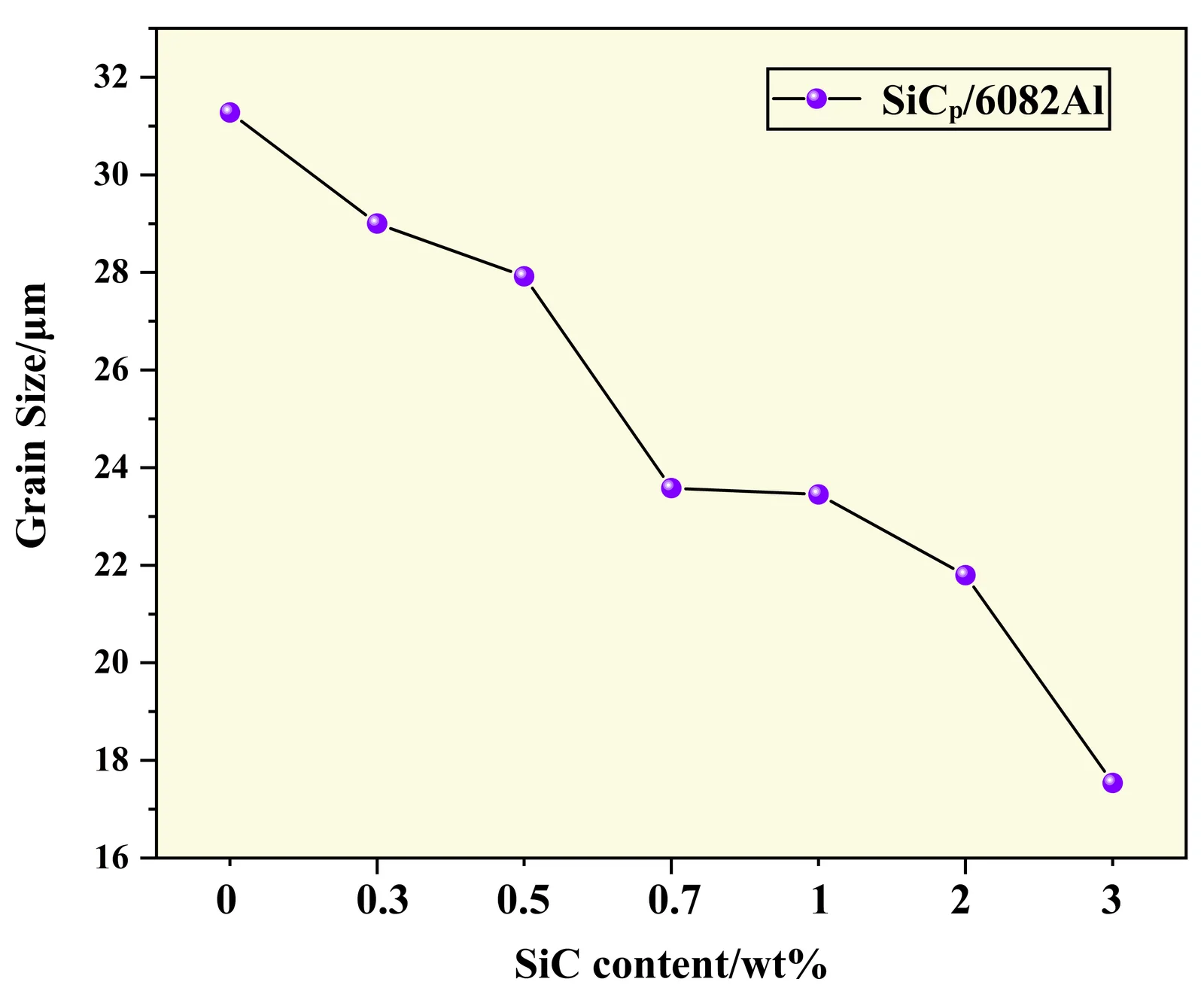
Mean grain size of SiC_p_/6082Al composites as a function of SiC content.

**Figure 7:**
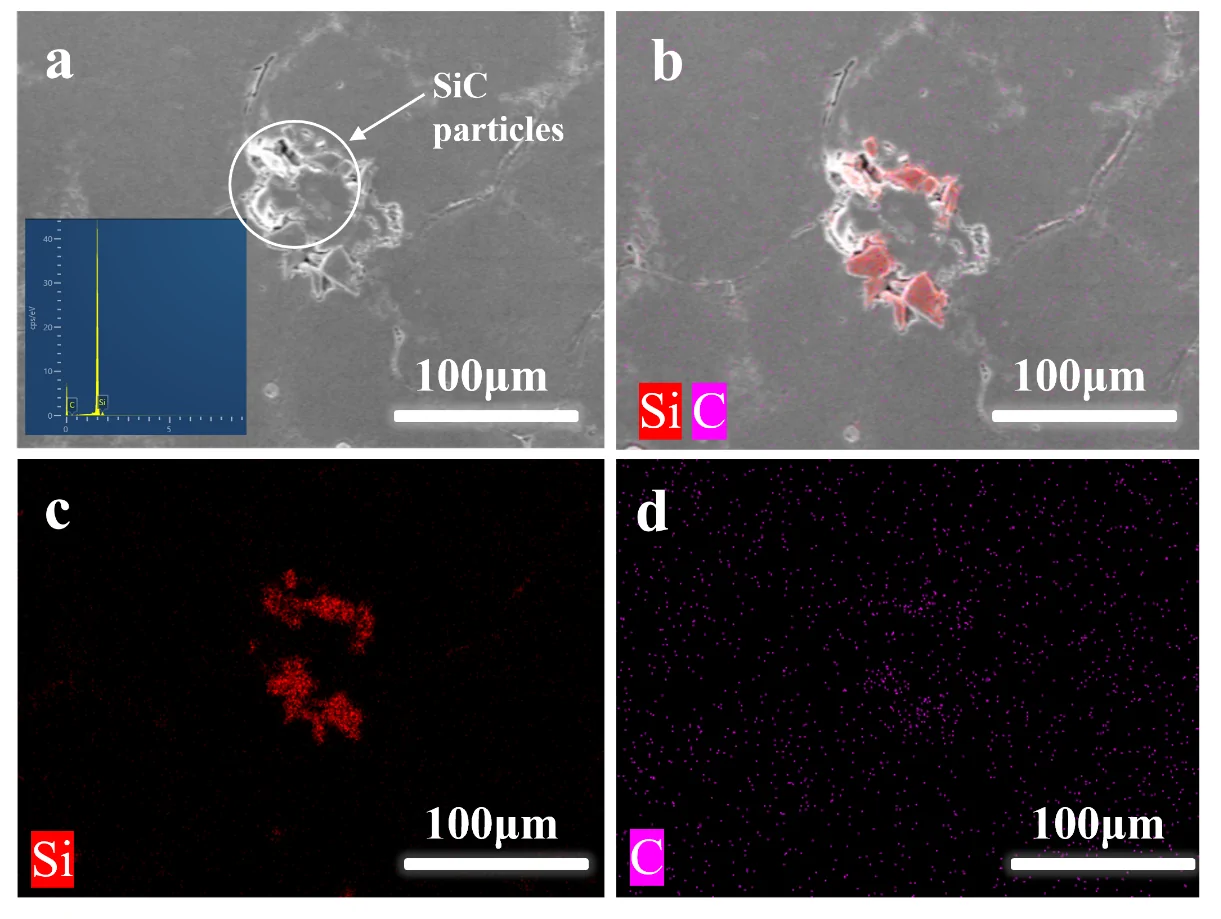
(**a**) SEM image of the 2 wt.% SiC_p_/6082Al composite and (**b**–**d**) corresponding combined, Si, and C EDS maps.

**Figure 8:**
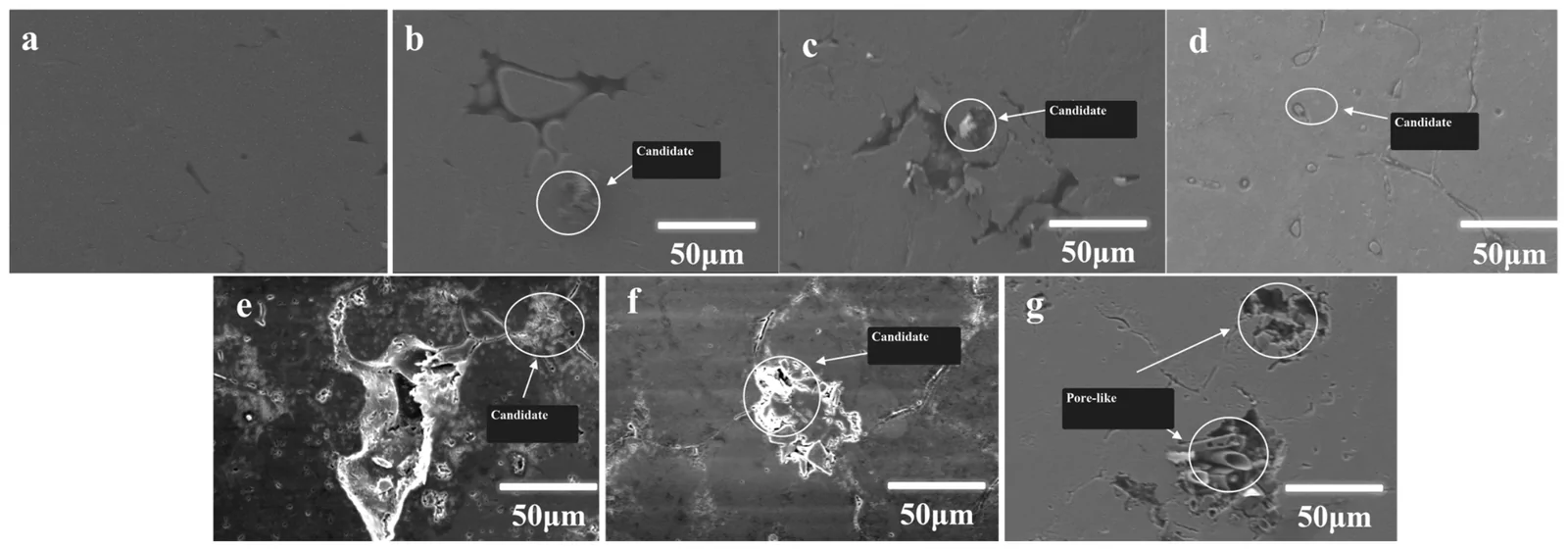
Representative SEM images of 6082 aluminum alloy and SiC_p_/6082Al composites containing (**a**) 0, (**b**) 0.3, (**c**) 0.5, (**d**) 0.7, (**e**) 1, (**f**) 2, and (**g**) 3 wt.% SiC. Annotations denote candidate particle-containing or pore-like regions and are not quantitative phase assignments.

**Figure 9:**
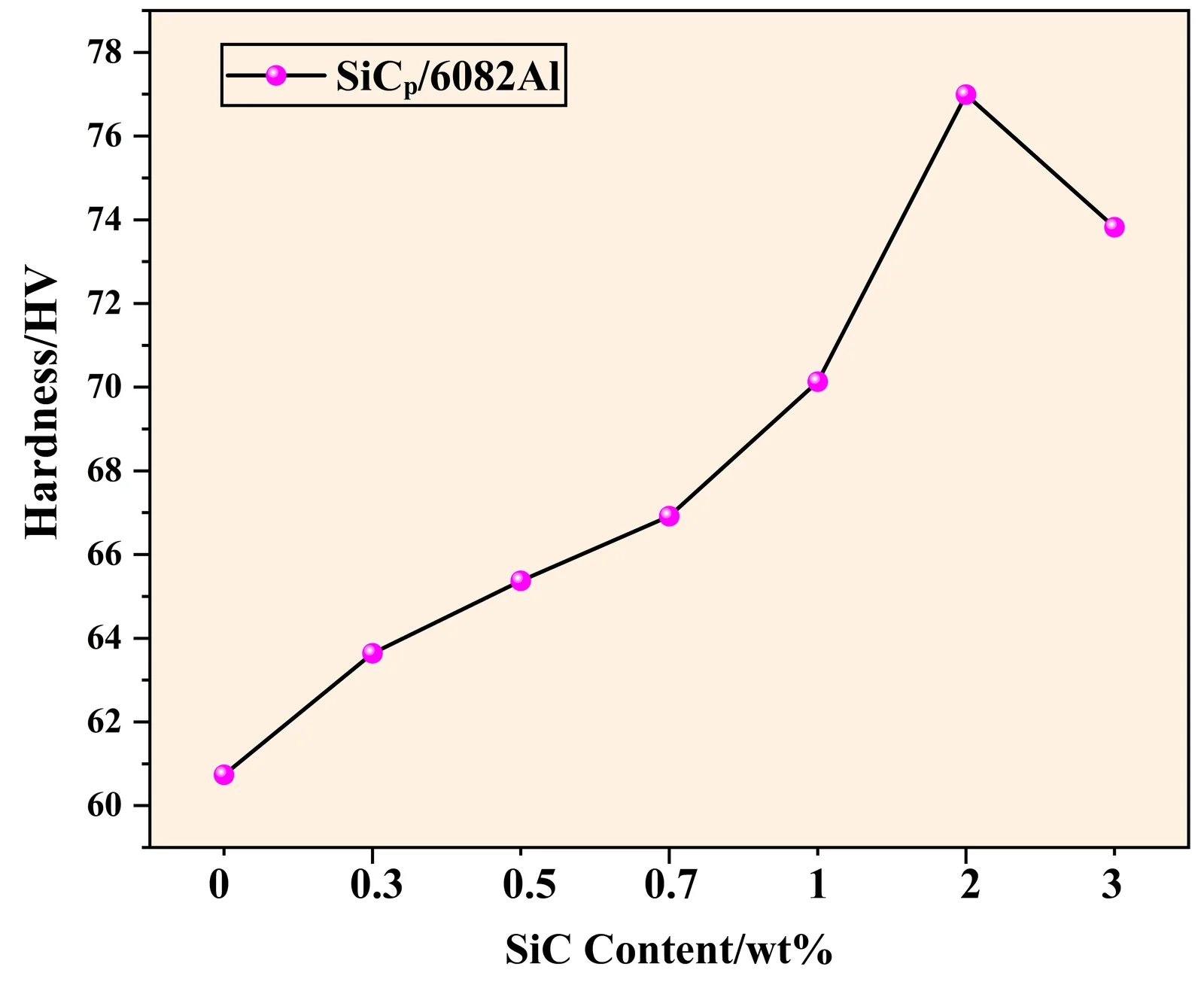
Mean Vickers hardness of SiC_p_/6082Al composites as a function of SiC content.

**Figure 12:**
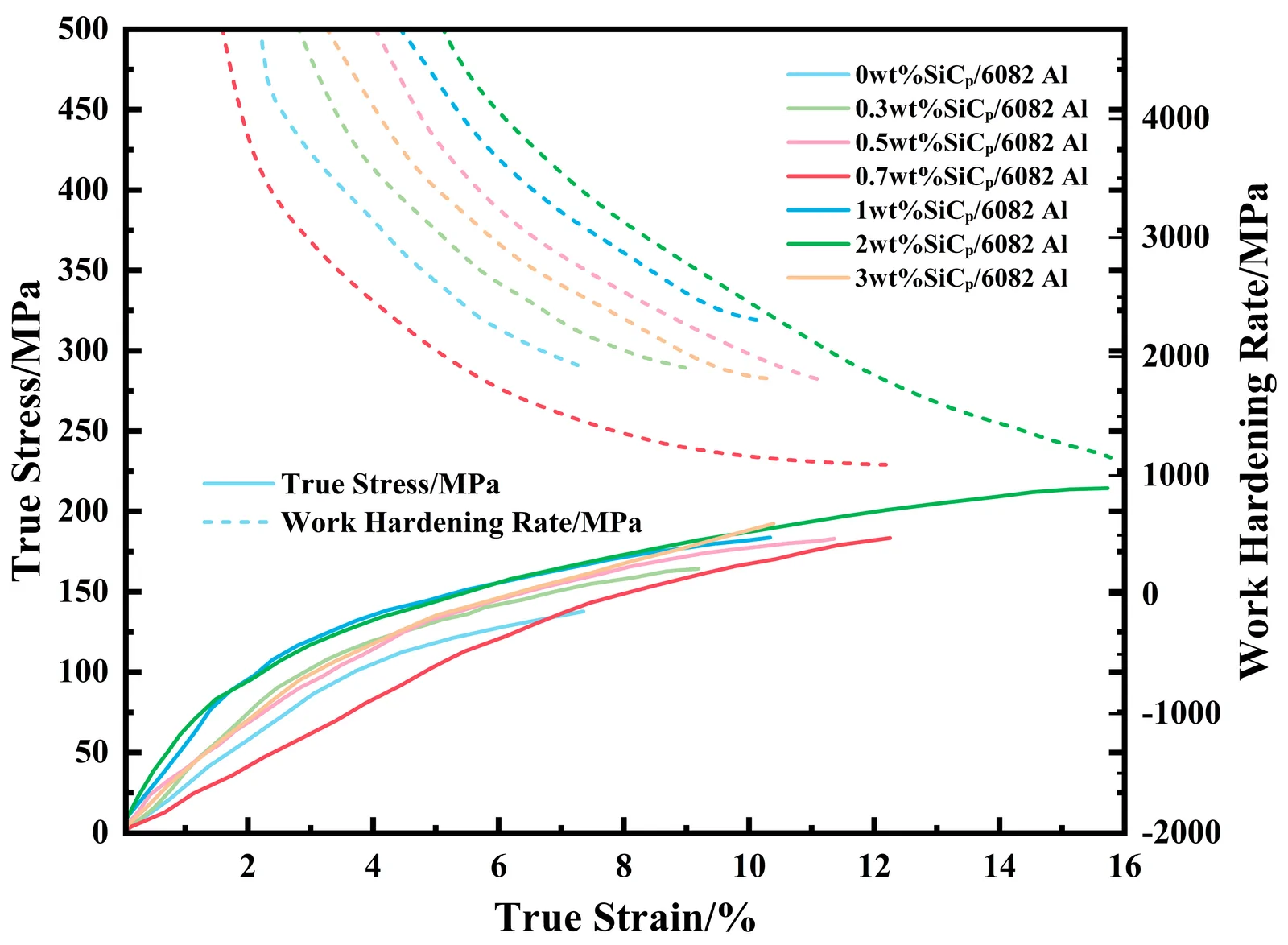
True stress–strain curves and work-hardening rates of SiC_p_/6082Al composites.

**Figure 13:**
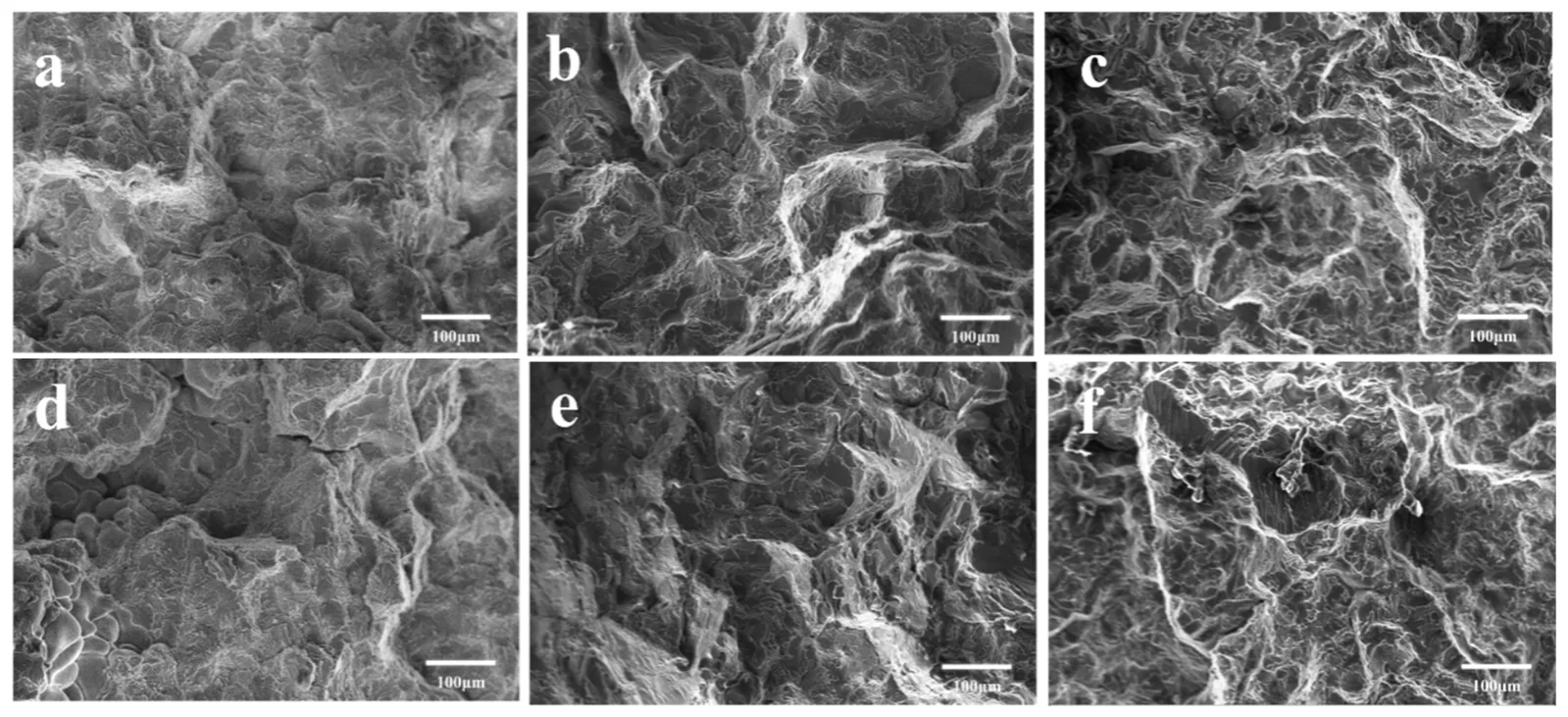
Tensile-fracture surfaces of SiC_p_/6082Al composites containing (**a**) 0.3, (**b**) 0.5, (**c**) 0.7, (**d**) 1, (**e**) 2, and (**f**) 3 wt.% SiC.

**Table 1:** Chemical composition documented for the starting 6082 alloy and measured mean tensile properties of the unreinforced control.

Chemical Composition (Mass Fraction, %)						Mechanical Properties (MPa)	
Si	Mg	Mn	Fe	Cu	Al	Tensile Strength	Yield Strength
0.7	0.6	0.41	0.11	0.03	Balance	128.33	70.58

## Data Availability

The processed mean values used in this study are included in the article. The original point-level hardness and grain-size records and the original work-hardening processing script are not available due to institutional intellectual property policies and laboratory regulations. The available XRD and mechanical-test records may be obtained from the corresponding author upon reasonable request.
